# The West Chicago Medical Society

**Published:** 1881-03

**Authors:** 


### ARTICLE XI.

THE WEST CHICAGO MEDICAL SOCIETY. Stated meeting, February 28, 1881. Prof. H. M. Lyman, M.D., read a Paper on Anæsthesia.

It comprised the history of alcohol, its chemistry and physiological action. Alcohol was extensively used in Europe, as a remedy, since the sixteenth century. Its general sale was authorized in 1676. The British soldiers became acquainted with it in Holland in 1551, and brought it to England. In 1774 it was generally used by the people of all classes in this country.

Alcohol coagulates albuminous substances, and, in large doses, acts as an irritant poison. It produces anæsthesia by diminishing the motion of the nerve-cells, and may in the same manner produce paralysis and death. In doses of one and a half ounces, it reduces the temperature, and in connection with quinine, it will produce a more permanent effect than when the latter is used alone.

These few notes show the wide range embraced by the Professor under the head of anæsthesia. This paper was only a part of a complete work on the subject, which he will have published in the course of the year.

A few questions were asked to and satisfactorily answered by Dr. Lyman. The name of Dr. G H. Peppitts was presented for membership and referred to the censors. Meeting adjourned.

